# Blood Vessels and Peripheral Nerves as Key Players in Cancer Progression and Therapy Resistance

**DOI:** 10.3390/cancers13174471

**Published:** 2021-09-05

**Authors:** Niccolò Roda, Giada Blandano, Pier Giuseppe Pelicci

**Affiliations:** 1 Department of Experimental Oncology, IEO, European Institute of Oncology IRCCS, 20139 Milan, Italy; Niccolo.Roda@ieo.it (N.R.); Giada.Blandano@ieo.it (G.B.); 2 Department of Oncology and Hemato-Oncology, University of Milan, 20122 Milan, Italy

**Keywords:** cancer progression, tumor microenvironment, blood vessels, peripheral nerves, metastasis, cancer therapy

## Abstract

**Simple Summary:**

The interactions between cancer cells and the surrounding blood vessels and peripheral nerves are critical in all the phases of tumor development. Accordingly, therapies that specifically target vessels and nerves represent promising anticancer approaches. The first aim of this review is to document the importance of blood vessels and peripheral nerves in both cancer onset and local or distant growth of tumoral cells. We then focus on the state-of-the-art therapies that limit cancer progression through the impairment of blood vessels and peripheral nerves. The mentioned literature is helpful for the scientific community to appreciate the recent advances in these two fundamental components of tumors.

**Abstract:**

Cancer cells continuously interact with the tumor microenvironment (TME), a heterogeneous milieu that surrounds the tumor mass and impinges on its phenotype. Among the components of the TME, blood vessels and peripheral nerves have been extensively studied in recent years for their prominent role in tumor development from tumor initiation. Cancer cells were shown to actively promote their own vascularization and innervation through the processes of angiogenesis and axonogenesis. Indeed, sprouting vessels and axons deliver several factors needed by cancer cells to survive and proliferate, including nutrients, oxygen, and growth signals, to the expanding tumor mass. Nerves and vessels are also fundamental for the process of metastatic spreading, as they provide both the pro-metastatic signals to the tumor and the scaffold through which cancer cells can reach distant organs. Not surprisingly, continuously growing attention is devoted to the development of therapies specifically targeting these structures, with promising initial results. In this review, we summarize the latest evidence that supports the importance of blood vessels and peripheral nerves in cancer pathogenesis, therapy resistance, and innovative treatments.

## 1. Introduction: The Heterogeneous Microenvironment of Tumors

Tumorigenesis represents a dynamic process that induces normal cells to lose their own specific identity and to acquire a series of malignant traits, including deregulated proliferation, evasion of apoptosis and immunosurveillance, and abnormal metabolism [1,2,3]. Notably, this process is driven by two main factors, namely, tumor-specific mutational burden, on one hand, and the continuous interaction of cancer cells with the surrounding stroma, on the other hand [4,5]. Stromal cells and the matrix in which they are embedded compose a heterogeneous ecosystem that has been referred to as the tumor microenvironment (TME) [4,5,6]. The communication between cancer cells and the TME relies on a dynamic molecular crosstalk involving both cell-to-cell interactions and secreted soluble factors (growth factors, cytokines, chemokines, vesicles, inflammatory mediators, and matrix remodeling enzymes) [4,5,6,7]. In this scenario, the TME continuously modifies the tumor phenotype and is critical to all the steps of cancer progression [8].

The cellular component of the TME comprises cancer-associated fibroblasts (CAFs), immune cells, blood vessels, and peripheral nerve terminals [4,5,6,7,8]. Both cancer and TME cells are embedded in the extracellular matrix (ECM), a connective structure that serves as a scaffold for the cells and regulates cell-to-cell crosstalk and a number of cancer phenotypes, including adhesion, migration, proliferation, and differentiation [9,10]. Notably, tumor cells interact with the ECM both during local progression and metastasis spreading. In this regard, matrix metalloproteinases (MMPs) are a group of endopeptidases which mediate the degradation of ECM components [11,12]. ECM degradation leads to the release of growth factors’ active form, thereby leading to increased proliferation in situ [12]. Moreover, MMP-mediated ECM degradation facilitates the invasion of cancer cells in the surrounding tissue, thereby facilitating the metastasis process [13].

CAFs are one of the most abundant stromal cells in the TME [14,15,16,17] and are involved in ECM secretion and remodeling [17,18], immune cell recruitment [19,20], and the maintenance of a pro-tumoral inflammatory microenvironment [21,22]. Immune cells include tumor-associated macrophages (TAMs) and tumor-infiltrating lymphocytes (TILs) [23,24,25]. TAMs are classified as M1 (classical activated macrophages) with antitumoral activity [26,27], and M2 (alternative activated macrophages) with pro-tumoral activity [26,28,29,30,31]. Ultimately, TILs infiltrate multiple solid tumors [24,25] and can accomplish either anti- or pro-tumoral effects [32]. For example, CD8+ cytotoxic T lymphocytes release interferon-γ (IFN-γ), which induces cancer cell death [33] and activates the aforementioned M1 macrophages [34]. The role of CD4+ T lymphocytes is, instead, more complex and can be either antitumoral [32,35,36] or pro-tumoral [32,36,37,38].

Furthermore, the TME is densely infiltrated by blood vessels and nerves [39,40,41,42,43,44], whose role in cancer pathogenesis, progression, and therapy resistance is the focus of the following sections of this review.

## 2. The Role of Blood Vessels and Peripheral Nerves in Tumor Initiation

Blood vessels and peripheral nerves represent a fundamental component of the TME, as they are involved in multiple phases of tumor development, from the early phases of tumorigenesis [45,46].

Blood vessels contribute to tumor initiation (i.e., the acquisition of the first mutations that ignite a tumoral phenotype) through the establishment of a prolonged inflammatory response [47]. Inflammation represents the protective response of a vascularized tissue to a damage signal (e.g., pathogenic infections, injuries, the presence of a foreign body). During inflammation, the blood vessels that perfuse the tissue undergo dilation, increased capillary permeability, and leukocyte extravasation [48]. An inflammatory microenvironment has been historically associated with tumorigenesis and, in particular, with the initiation process [47,48,49]. A large body of experimental evidence suggests that the leukocytes that invade the tissue from the bloodstream foster the release of reactive oxygen species (ROS) and reactive nitrogen intermediates (RNI) that, in turn, promote DNA damage and genomic instability [50,51]. As an example, the strong association between the inflammatory microenvironment and cancer initiation was demonstrated in gastric cancer (GC) development following *H. pylori* infection [52,53]. In patients infected by *H. pylori*, leukocytes infiltrating the gastric mucosa upregulate the inducible nitric oxide synthase, ultimately increasing the production of RNI [54]. In line with this, 8-hydroxydeoxyguanosine, a marker for oxygen free radical-induced DNA damage, was shown to accumulate in the patients’ gastric epithelium upon *H. pylori* infection [55].

In parallel, peripheral nerves display a major role in the first steps of tumorigenesis [46,56]. Pioneer studies on spontaneous mouse models of prostate cancer (PC) showed that either the denervation of adrenergic nerves or the inhibition of adrenergic signaling in the prostate significantly delays tumor formation [57]. Similarly, the depletion of doublecortin-expressing neural progenitors in the central nervous system hinders the development of spontaneous PC in vivo [58]. In the case of spontaneous pancreatic ductal adenocarcinoma (PDAC), increased release of catecholamines by sympathetic neurons in the pancreas fosters the development of neoplastic lesions through the activation of the β-adrenergic receptor. Indeed, β-adrenergic signaling stimulates the phosphokinase A (PKA) and mitogen-activated protein kinase (MAPK) pathways in pancreatic cells [59]. In line with this, a recent study on a distinct mouse model of spontaneous PDAC demonstrated that ablation of sensory neurons significantly hampers tumor development [60]. Interestingly, the authors showed that neurons promote PDAC development through the establishment of neurogenic inflammation (i.e., an inflammatory condition mediated by the release of neurotrophic factors) in the pancreatic microenvironment [60,61]. Furthermore, in a spontaneous model of GC, either surgical or pharmacological denervation of the stomach markedly reduces the incidence of GC through the inhibition of the Wnt and Notch signaling pathways in the gastric epithelium [62]. Finally, the surgical ablation of sensory cutaneous nerves in hair follicles inhibits tumorigenesis in a spontaneous model of basal cell carcinoma, with tumors decreasing both in size and abundance upon denervation [63]. This antitumoral effect is due to the arrest of paracrine neuronal signals: indeed, cutaneous sensory nerves were proven to release the ligands Sonic hedgehog, Desert hedgehog, and Indian hedgehog, which activate the hedgehog signaling pathway in epithelial cells and promote tumorigenesis [63].

## 3. Angiogenesis and Axonogenesis

**Figure 1:**
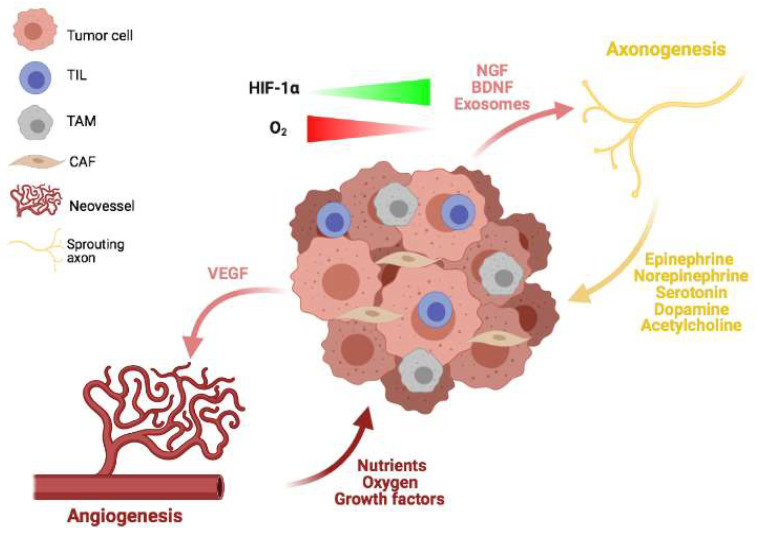
Angiogenesis and axonogenesis in the TME. The innermost core of the tumor experiences a progressive decrease in oxygen pressure, which leads to the HIF-1α-mediated expression of VEGF. This soluble factor stimulates the abnormal sprouting of blood vessels, which restore optimal levels of oxygen, nutrients, and growth factors in the mass (angiogenesis). In parallel, growth factors and exosomes are released by tumoral cells and target the nerve terminals in the surrounding tissue, leading to axon sprouting within the TME (axonogenesis). Nerve terminals interact with the expanding mass through the release of neurotransmitters (e.g., epinephrine, norepinephrine, serotonin, dopamine, and acetylcholine), thereby shaping the local and metastatic tumor progression.

Blood vessels and peripheral nerves contribute to all the phases of cancer progression. Due to this, tumoral cells establish a precisely fine-tuned molecular network to communicate both with vessels and nerves from the early stages of tumorigenesis (Figure 1). Since the 1970s, the importance of cancer vascularization was pointed out as tumors cannot grow beyond a small volume (indicatively 1–2 mm^3^) in the absence of perfusion [45]. Therefore, cancer cells need to induce their own vascularization to obtain both oxygen and nutrients that simple diffusion cannot provide in a sufficient quantity [64].

The process through which cancer cells promote the sprouting of new vessels is referred to as angiogenesis [65,66] and represents a key hallmark of both solid and hematologic malignancies [67]. In this respect, the progression from a non-angiogenic to an angiogenic phenotype has been defined as the angiogenic switch and is regulated by oxygen levels [68]. Indeed, deregulated growth of cancer cells generates tumor masses which are progressively more and more distant from local capillaries. For this reason, the diffusion of oxygen becomes inefficient, and the innermost core of the tumor undergoes a condition of hypoxia. The master regulator of the hypoxia response in cells is the transcription factor hypoxia-inducible factor 1α (HIF-1α), whose turnover is tightly regulated by intracellular oxygen levels. In normoxic conditions, HIF-1α undergoes oxygen-dependent prolyl-hydroxylation, and hydroxylated HIF-1α is targeted to proteasomal degradation. However, in hypoxia, the oxygen-dependent hydroxylation is not efficient, and HIF-1α forms a heterodimer with HIF-1β and regulates the expression of several target genes [68,69]. One of the main regulators of hypoxia-induced angiogenesis is the vascular endothelial growth factor A (VEGF-A or simply VEGF), a member of the platelet-derived growth factor family that induces the proliferation, migration, and fenestration of capillaries, thus providing a new oxygen supply to the tumor [70,71,72].

Interestingly, angiogenesis is not an exclusive trait of solid tumors, as several hematologic malignancies were reported to upregulate this phenotype [67]. In particular, the leukemic niche of acute myeloid leukemia (AML) displays an increased vascular density as compared to normal bone marrow [73,74]. In this respect, the increased vascularity of AML and myelodysplastic syndromes correlates with an increased expression of VEGF in leukemic cells [75]. Intriguingly, increased secretion of VEGF in AML cells was shown to activate an autocrine VEGF-VEGFR2 signaling pathway which leads to increased blast proliferation [76].

Beside angiogenesis, tumor cells promote neurogenesis (increased neuron number) and axonogenesis (increased axon sprouting in the TME) during tumor progression [43,77,78]. The pioneer work of Rowley and colleagues demonstrated that PC patients display both an increased nerve density in pre-neoplastic lesions and increased neurogenesis in prostatic ganglia. Moreover, PC cells were shown to induce axon sprouting within the tumor mass. Notably, the upregulation of semaphorin 4F in PC cells was shown to be sufficient to induce both neurogenesis and axonogenesis [79]. Similarly, PDAC patients display copy number variations in several genes involved in axon guidance pathways, including semaphorins, further suggesting a role for this gene family in cancer progression [80]. Mechanistically, several works demonstrated that cancer cells promote neuronal outgrowth through the paracrine release of growth factors, a phenomenon known as the neurotrophic effect. For example, the study of Hondermarck and colleagues revealed that breast cancer (BC) cells overexpress the nerve growth factor (NGF) in vitro, which induces neurite sprouting from co-cultured sympathetic neurons [81]. Similarly, other studies on BC reported that both NGF [82,83] and VEGF [83] released from tumoral cells foster axon infiltration in vivo. Similar results were obtained in PC cells in vitro, with the overexpression of the NGF precursor leading to axonogenesis of co-cultured neuronal cells [84]. Interestingly, a study on PC in vivo showed that prostate metastases in the bones are innervated by nociceptors in an NGF-dependent mechanism. However, the authors did not find NGF in the tumoral cells, thereby suggesting a role for inflammatory, immune, and stromal cells in releasing the growth factor [85]. Analogously, a recent in vivo work on ovarian cancer (OC) demonstrated that cancer cells stimulated by norepinephrine (released by sympathetic neurons) increase the expression of the brain-derived neurotrophic factor (BDNF), whose release fosters tumor innervation [86]. This last work suggests a sustained feed-forward loop in which adrenergic stimulation of OC cells induces tumoral cells to promote their own innervation.

In addition to the secretion of soluble factors, recent studies investigated the role of exosomes in promoting axonogenesis. Exosomes are nano-sized vesicles generated from late endosomes and released by cells into the surroundings upon fusion of multivesicular bodies and the plasma membrane. Exosomes are highly complex structures which were shown to contain hundreds of distinct lipids and thousands of different proteins (including tetraspanins, heat shock proteins, and proteins involved in membrane transport and fusion) and RNAs (both mRNAs and miRNAs). Exosomes represents an intercellular communication mechanism and influence the behavior of target cells as a result of the exosome-specific cargo of proteins, nucleic acids, and lipids [87]. Vermeer and colleagues reported that head and neck squamous cell carcinoma (HNSCC) and oropharyngeal squamous cell carcinoma increase axonogenesis in vitro and in vivo through the release of EphrinB1-enriched exosomes [88]. Likewise, cervical cancer cells were proven to stimulate axonogenesis through the release of exosomes [89]. Finally, a recent study on p53-deficient oral cavity squamous cell carcinoma demonstrated that cancer cells release miRNA-enriched extracellular vesicles which stimulate the differentiation of sensory nerves into adrenergic neurons and the sprouting of these neurons [90].

Interestingly, while solid tumors are reported to induce neurogenesis and axonogenesis, works on hematologic malignancies revealed a significant depletion of neuron terminals in the leukemic stem cell niche. Indeed, the sympathetic nerve fibers that physiologically innervate the hematopoietic stem cell niche [91] progressively undergo degeneration in myeloproliferative tumors: mechanistically, cancer cells produce interleukin-1β, which, in turn, induces neural cell degeneration [92]. Coherently, a recent study on AML showed that cancer cells rapidly disrupt sympathetic neurons in the niche in order to deplete niche cells that maintain a healthy hematopoiesis [93]. Therefore, while angiogenesis represents a common trait of both solid and hematologic malignancies, axonogenesis is fostered upon solid tumor growth and inhibited by the development of hematologic cancers.

## 4. The Role of Blood Vessels and Peripheral Nerves in Local Tumor Progression

Several works focused on models of tumor growth in the absence of vessels [94,95]. In the avascular phase, cancer cells proliferate, with only peripheral cells experiencing a normoxic condition. The innermost core, instead, progresses from an initial phase of quiescence, due to oxygen shortage, to a necrosis phase in anoxia, where dead cells are constantly removed by actively proliferating cells [94,95]. Notably, in this situation, the tumor cannot grow indefinitely, with peripheral cells expanding the tumor radius linearly (and the volume cubically as a consequence). Rather, tumoral masses progress from a linear growth phase towards a plateau phase, where the number of dying cells equates to the number of proliferating cells [95]. Therefore, tumors need to proceed towards a vascular phase to scale up in terms of volume. Cancer cells ignite angiogenesis and new sprouting vessels provide oxygen and nutrient supply to sustain tumor growth [96,97,98]. When angiogenesis is triggered, quiescent cells re-enter the cell cycle, and the tumor volume increases accordingly [94]. Indeed, new sprouting vessels are critical for tumor growth as they ensure the supply of oxygen and nutrients [99,100,101] (including glucose [102], amino acids [103], lipids [104], hormones, and growth factors [105,106,107]).

While blood vessels promote local tumor growth through the supply of nutrients and oxygen, peripheral nerves foster cancer proliferation through the release of neurotransmitters [108]. The role of peripheral nerves in promoting cancer progression in situ was historically demonstrated via denervation experiments, which showed that surgical denervation and pharmacological denervation lead to, respectively, GC [62] and BC [109] regression in vivo.

The catecholamines epinephrine and norepinephrine were associated with local tumor growth in several cancer models [110]. Pioneer works on PC demonstrated that adrenergic signals in the prostate are involved both in tumor development and growth in situ [57]. Similarly, a recent study on PDAC revealed that sympathetic neuron-mediated catecholamine signaling leads to increased NGF expression in PDAC cells, which, in turn, fosters both tumor proliferation (via autocrine signaling) and axonogenesis in vivo [59]. Moreover, adrenergic signals stimulate tumor growth by promoting angiogenesis [108]. In this regard, a study on OC demonstrated that chronic behavioral stress, which results in increased tissue catecholamine levels, leads to increased tumor growth in vivo. This effect is mediated by the tumoral β2-adrenergic receptor, which, in turn, regulates the cyclic AMP (cAMP)–PKA signaling pathway. Notably, this pathway promotes the expression of VEGF in tumoral cells, thus increasing tumor vascularization [111]. Interestingly, the endothelial β2-adrenergic receptor was found to be relevant for the angiogenic switch: when PC cells are injected in mice lacking this receptor, tumors persist in the avascular phase, and their growth is arrested. Mechanistically, endothelial cells rely on glycolytic metabolism to undergo angiogenesis: the inhibition of β2-adrenergic signaling enhances the oxidative phosphorylation, which dampens the angiogenesis process [112]. Concordantly with these results, the surgical denervation of adrenergic nerve fibers was shown to abrogate BC growth in vivo [113].

Interestingly, while the catecholamines epinephrine and norepinephrine were historically associated with increased tumor growth, their precursor dopamine generally exerts the opposite effect through blocking the angiogenic switch [108]. Dopaminergic neuron-released dopamine, indeed, induces the internalization of VEGF receptor 2 in endothelial cells, thereby preventing the angiogenesis process in vivo [114,115]. At the same time, dopamine was shown to decrease the mobilization of endothelial progenitor cells from the bone marrow to peripheral tissues, thereby preventing their incorporation in newly formed vessels [116,117]. Concordantly, dopamine administration was reported to revert the norepinephrine-induced angiogenic switch in OC in vivo through the reduction in cAMP levels in endothelial cells and through the inhibition of the VEGF-mediated signaling pathway [118]. The effect of dopamine on endothelial VEGF receptor 2 internalization was also reported in GC [116,117] and melanoma [114,115], with a decreased tumor volume as a consequence. In line with these findings, ablation of dopaminergic nerves was proven to promote tumor progression in GC [117], sarcoma [116], and melanoma [114,115]. Oppositely, a recent work on glioblastoma (GBM) revealed that dopaminergic neuron-released dopamine markedly stimulates tumor growth. Indeed, the antagonists of dopamine receptor D4 block the downstream MAPK and mTOR pathways and inhibit autophagy completion in GBM cells in vivo, leading to autophagic vacuole accumulation and apoptosis [119]. Therefore, the effects of dopamine on primary tumor growth largely depend on the tumor type.

In addition to catecholamines, other neurotransmitters were shown to play a role in promoting primary tumor growth [107]. For example, serotonin (released by serotoninergic neurons) was reported to induce hepatocellular carcinoma (HCC) growth in vivo through the inhibition of autophagy [120], and to foster colorectal cancer (CRC) progression by inducing the angiogenic switch [121]. Mechanistically, serotonin reduces levels of MMP-12 in TAMs, thereby lowering the levels of macrophagic angiostatin (an angiogenesis inhibitor) [121]. Other works pointed out the role in tumor progression of acetylcholine, a neurotransmitter released by parasympathetic cholinergic nerve fibers [108]. Similar to dopamine, acetylcholine reduces tumor growth. Indeed, cholinergic stimulation of BC in vivo dampens tumor growth through the suppression of immune checkpoint molecules in tumoral cells [113]. Similar results were obtained in two independent studies on PDAC [59,122], where cholinergic stimulation of PDAC cells in vivo suppresses the MAPK and PI3K-Akt pathways, leading to the depletion of cancer stem cells [59]. Notably, PDAC cells were shown to express high levels of acetylcholinesterase—the enzyme that degrades acetylcholine—whose downregulation reduces tumor growth in vivo through inhibition of both the MAPK pathway and TAM recruitment [122]. Intriguingly, a recent work on GC revealed that acetylcholine agonists promote cancer growth in vivo through the activation of epidermal growth factor (EGF) receptor signaling, which activates the MAPK and PI3K-Akt pathways [123]. Therefore, similar to dopamine, the acetylcholine effects on tumor growth are complex and highly dependent on the model.

## 5. Blood Vessels and Peripheral Nerves in Metastatic Progression

Beside their role in promoting in situ tumor growth, blood vessels and peripheral nerves are involved in the spreading of metastatic cells [62,124]. The process of metastasization is extremely inefficient, with only 0.01% of metastatic cells managing to generate a distant metastasis [125]. However, tumor masses shed into the circulation several millions of cells every day, thus increasing the risk of metastasis formation [126]. In this scenario, newly formed vessels originating upon the angiogenic switch are exploited by tumoral cells to reach distant organs [127]. Notably, these vessels are leaky, immature, tortuous, and highly permeable [127,128]: therefore, while not perfectly suited for the delivery of oxygen and nutrients to the surrounding TME, they can be easily invaded by tumoral cells [129].

Expectedly, a huge number of studies showed a strong inverse correlation between an increased microvessel density (MVD) in tumors and patient prognosis [130,131]. One of the first works that correlated the MVD with the spreading of fibrosarcoma metastatic cells in vivo was performed by Saidel and colleagues in the 1970s. The authors showed that higher values of MVD correspond to higher numbers of tumoral cells that reach the circulation and therefore metastasize [132]. Afterwards, several clinical studies investigated the correlation between MVD and metastatic burden in patients. A pioneer study on melanoma patients revealed that the MVD value was significantly higher in patients that experienced metastases than in patients that did not [133]. This result was later confirmed by Kaner and colleagues, who demonstrated that melanoma metastasis spreading positively correlated both with MVD and with the increased expression of VEGF in tumor cells [134]. Analogously, several studies pointed out the same relationship in BC patients, where tumors display a higher MVD value than normal breast tissue [135]. In this regard, higher values of MVD are predictive of BC metastasis development and significantly worse prognosis [135,136,137]. Similar results were obtained also in PC [138], non-small cell lung cancer (NSCLC) [139], GC [140], and others [127,130].

While blood vessels contribute to the metastatic progression by providing the path towards the circulation, peripheral nerves impact the metastasization process in multiple ways [42,124,141,142]. Increased neurogenesis was associated with poor patient outcome, enhanced metastatic burden, and higher tumor grade in several models, including CRC [143], BC [144,145], PDAC [146], and thyroid cancer [147]. In this respect, catecholamines were shown to significantly enhance metastasis spreading [58,147]. For example, chronic stress was reported to foster PDAC growth and dissemination in tissues adjacent to the pancreas in vivo. Notably, this latter effect is mediated by adrenergic signals that upregulate the expression of invasion-related genes (e.g., MMPs) [148]. Similarly, norepinephrine administration enhances CRC migration properties in vitro through the activation of protein tyrosine kinase of the *src* family [149]. Coherently, two independent works on BC revealed that adrenergic signals from sympathetic nerve fibers both increase the migration of BC cells in vitro [150] and foster metastasis spreading in vivo [151]. While the pro-migratory effects were shown to be tumor cell-autonomous, the pro-metastatic phenotype is due to an increased norepinephrine-dependent TAM recruitment in the tumor mass. The recruited TAMs overexpress genes involved in immune suppression (e.g., Transforming growth factor β and Arg1) and in metastasis spreading (e.g., VEGF, MMP9) [151]. The role of norepinephrine in promoting metastasis spreading was further confirmed in PC, where catecholamine administration increases the number of metastatic foci in lymph nodes. Notably, this metastatic phenotype depends on the induction of the epithelial–mesenchymal transition (EMT) [152]. Ultimately, OC cells exposed to either norepinephrine or epinephrine display enhanced proliferation and reduced sensitivity to anoikis: these effects are mediated by the focal adhesion kinase (FAK) signaling, and, indeed, OC patients with increased levels of FAK display a significantly worse overall survival [153].

In addition to the effects on tumoral—and stromal [151]—cells, neurotransmitters foster metastasis spreading by exacerbating the angiogenic phenotype. As discussed in the previous chapter, neurotransmitters promote the angiogenic switch, thereby leading to enhanced tumor growth in situ [111,121]. Concordantly, independent works confirmed that adrenergic stimulation of BC [154,155] and OC [156] leads to increased production of VEGF in vitro. Likewise, catecholamines lead to the overexpression of VEGF and MMPs in GC cells, thus fostering a pro-angiogenic and pro-metastatic phenotype [157].

Eventually, peripheral nerves contribute to metastasis spreading by providing the structural scaffold for cancer cells to reach distant organs. Perineural invasion (PNI) indicates the process through which cancer cells invade the peripheral nerves and spread towards distant organs [158,159]. Notably, this metastatic route can be observed both in the presence and in the absence of vascular or lymphatic invasion [158]. In PNI, cancer cells spread along and within the neural sheath of nerves, and their migration is regulated by the action of growth factors (e.g., NGF, BDNF) that are involved in axon guidance [158,159]. A pivotal study by Van Landeghem and colleagues revealed, indeed, that CRC cells interact with enteric nervous system neurons in vitro, thereby suggesting that these neurons play a role in guiding the migration of metastatic CRC cells. Notably, this interaction is partly mediated by the homophilic interaction between tumoral and neuronal N-cadherins and partly by the heterophilic interaction between neuronal L1CAM and tumoral integrins [160]. The PNI route was reported for several cancer types and is generally associated with reduced overall survival of patients [159]. For example, several works on CRC revealed that PNI positively correlates with tumor grade, invasive behavior, lymph node metastases, and reduced overall survival [161,162,163,164]. Similar results were reported in PDAC [165], PC [166,167], oral tongue squamous cell carcinoma [168], and GC [169,170], while no association between overall survival and PNI was scored in invasive BC [158,171], despite a significantly increased risk in locoregional relapse [172]. Mechanistically, in PDAC, PNI is mediated by the cooperation between glial cells, nerves, and cancer cells. Glial cells secrete the glial cell line-derived neurotrophic factor (GDNF) that acts as a chemoattractant towards nerves. In parallel, nerves release a soluble form of GDNF family receptor (GFR)α1, which acts as a coreceptor for tumoral Ret proto-oncogene (RET) receptors: upon GFRα1 release, tumoral cells can respond to GDNF and efficiently perform PNI [173]. Notably, a similar mechanism was also reported in BC [174] and bile duct carcinoma [175]. Therefore, a sustained molecular crosstalk between tumoral cells and nerve components is necessary to promote PNI. Instead, a recent study on HNSCC revealed that the neuropeptide galanin acts on cancer cells and fosters their migration through the upregulation of cyclooxygenase-2. In addition, stimulated HNSCC overexpress galanin, which promotes axonogenesis: this feed-forward loop allows the nerve invasion of cancer cells [176]. Besides PNI, HNSCC cells were shown to spread macroscopic extension along peripheral nerves (mostly trigeminal and facial nerves), a process referred to as perineural tumor spread (PNTS) [177]. Notably, PNTS was reported to severely impair patient quality of life (increased neuropathic pain, numbness, and paralysis) and to worsen patient prognosis [178,179,180]. Despite being macroscopic, PNTS can be overlooked in imaging analysis: as up to 40% of patients display an asymptomatic PNTS, clinicians should be particularly careful when evaluating patient imaging studies [177].

## 6. Blood Vessels and Peripheral Nerves in Cancer Treatment

### 6.1. Blood Vessels

In light of its paramount role in cancer progression, angiogenesis represents a promising target in anticancer therapies. Several pre-clinical models showed that antiangiogenic therapies markedly reduce the growth of both primary and metastatic tumors [127]. For example, a work by Hanahan and colleagues demonstrated a significant reduction in islet cell carcinoma volume upon angiogenesis pharmacological inhibition: notably, while the proliferative index between treated and untreated tumors was comparable, angiogenesis inhibition strikingly increased the apoptosis of tumoral cells [181]. Similar results were obtained in in vivo models of glioma, where the inhibition of angiogenesis resulted in decreased tumor growth and enhanced apoptotic cell death as well [182]. Coherently, angiogenesis inhibition was shown to disrupt the tumor vasculature, increase apoptosis, and suppress in vivo tumor growth in several pre-clinical models of murine and human tumors [183,184]. In line with this, an in vivo study on CRC showed that the administration of regorafenib—an inhibitor of endothelial VEGF receptor—markedly suppresses primary tumor growth and prevents the formation of distant liver metastases [185]. Similar results were replicated by De Palma and colleagues in mammary and pancreatic carcinomas, where angiogenesis inhibition prevents spreading of metastasis from in situ growing tumors and outgrowth of metastases upon distant organ seeding [186]. The usage of angiogenesis inhibitors alone, however, is limited by their intrinsic inability to eradicate tumors [187]. Consistently, treated tumors were shown to restart growing upon antiangiogenic therapy withdrawal [188]. Importantly, antiangiogenic therapies were reported to have relevant side effects, especially for the nervous system. In particular, the alteration of blood vessels’ physiology leads to both an increased risk of thromboembolic events [189] and mild to severe hemorrhage in the brain [190]. Furthermore, several patients were shown to develop posterior reversible encephalopathy syndrome (PRES) upon antiangiogenic therapy. PRES clinical symptoms involve headache, nausea and emesis, visual loss, and seizures [191]. Although most of these symptoms are reversible, the onset of secondary cerebral ischemia or bleeding may lead to permanent neurological disability [192]. Nevertheless, antiangiogenic treatments have become part of current anticancer strategies and were shown to improve the prognosis of oncological patients [193].

**Figure 2:**
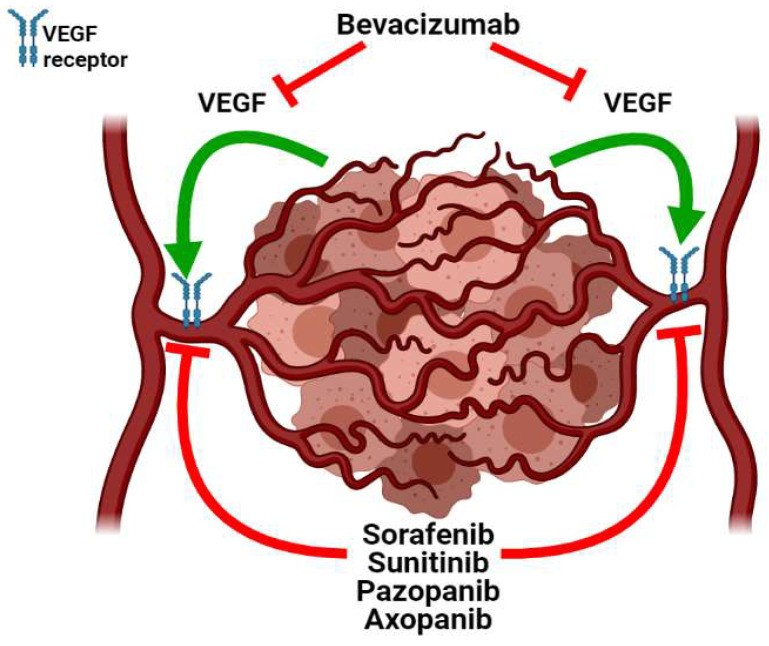
Antiangiogenic therapies aimed at the normalization of the tumor vasculature. Tyrosine kinase inhibitors (namely, sorafenib, sunitinib, pazopanib, and axopanib) hinder the abnormal tumor vasculature by inhibiting the VEGF receptor signaling, which, in turn, prevents endothelial migration towards the TME. On the other hand, the anti-VEGF monoclonal antibody bevacizumab counters tumor-induced angiogenesis by sequestering VEGF, thus impeding its binding to endothelial VEGF receptor.

Indeed, the disruption of the non-physiological tumor vasculature represents a key step in cancer eradication, as initially proposed by R. K. Jain with the concept of the normalization of the tumor vasculature. Angiogenesis generates a blood vessel network where capillaries are leaky and tortuous, thereby resulting in increased interstitial pressure and abnormalities in tumor perfusion [194,195]. These characteristics of the tumor vasculature have detrimental effects, such as the formation of dangerous edemas in the tumor mass [193]. At the same time, the increased permeability of the vasculature coupled with the impaired lymphatic drainage enhances the retention of high-molecular weight drugs in solid tumors (enhanced permeability and retention (EPR) effect). As a result of the EPR, high-molecular weight drugs accumulate more in the tumor mass than in the other tissues. Unfortunately, the high interstitial pressure of the tumor center counters the EPR effect, leading to increased retention of antitumor drugs only in the periphery of the mass [196,197]. In addition, the blood flow was demonstrated to sharply drop in the tumor center as compared to the periphery [198]. This drop increases the viscosity of the blood, which, in turn, reduces the efficiency of the drug distribution in the mass [199]. On top of that, the large inter-capillary distance within the tumor mass [198] obstructs the diffusion of drugs, which therefore achieve a therapeutic concentration only in the near proximity of vessels [194,200]. In this scenario, several cells within the mass experience inefficient perfusion even upon the angiogenic switch, and this condition makes them resistant to therapies [201]. Consequently, the tumor vasculature contributes to chemoresistance as the initial drug-mediated regression is followed by a relapse sustained by the residual cells [196]. Therefore, the normalization of the tumor vasculature through a precise angiogenesis inhibition would destroy the abnormal vessels, while the normal, physiological vessels are retained (Figure 2). This normalization improves the drug distribution in the TME and enhances the efficiency of anticancer treatment [202].

With respect to angiogenesis-associated edema, several works on GBM pointed out that normalization of the tumor vasculature significantly improves patient survival. Angiogenesis inhibition was proven to alleviate intra-tumoral edema [203], with reduced intracranial pressure and a significantly improved prognosis as a consequence [204,205]. In addition, antiangiogenic therapies were shown to improve the drug distribution within the TME. A pioneer study by Jain and colleagues showed that a normalized tumor vasculature results in better penetration of molecules in the tumor [206]. Likewise, two independent studies on glioma [207] and CRC [208] revealed that vascular normalization improves the distribution of chemotherapeutic drugs in the tumor interstitial fluid [207] and drug uptake by cancer cells [208]. The effects of the normalization of the tumor vasculature were mirrored in the presence of radiotherapy. Inhibition of angiogenesis enhances oxygenation of certain regions of the tumor [208], which, in turn, improves the efficacy of radiations. In this regard, the use of antiangiogenic drugs was shown to produce a time window of improved tumor oxygenation, in which radiotherapy is more effective [209]. Similarly, the parallel administration of antiangiogenic therapy and radiotherapy significantly decreases PC growth in vivo as compared to radiotherapy alone [210].

Several inhibitors of the VEGF receptor signaling (e.g., sorafenib, sunitinib, pazopanib, axopanib) have been developed in recent years to counter tumor-associated angiogenesis. These small molecules are referred to as tyrosine kinase inhibitors (TKIs) as they block the tyrosine kinase domain of the VEGF receptor, thereby preventing intracellular signaling and endothelial cell migration [193]. The oral administration of TKIs was demonstrated to improve patient prognosis in clinical trials. Sorafenib was shown to significantly ameliorate the progression-free survival of clear cell renal cell carcinoma (ccRCC) patients [211] and the overall survival of HCC patients [212]. Sunitinib treatment induced a significant increase in progression-free survival in ccRCC patients [213] and overall survival in pancreatic neuroendocrine tumor patients [214]. The new VEGF receptor inhibitors axopanib and pazopanib were shown to be less toxic [215] and to induce prolonged progression-free survival [216] as compared to sorafenib and sunitinib. In addition to TKIs, another branch of antiangiogenic therapy relies on the use of monoclonal antibodies capable of binding and neutralizing soluble VEGF [193]. One of the most important anti-VEGF antibodies is bevacizumab, whose role was first elucidated by Jain and colleagues in 2004 [207]. Intravenous administration of bevacizumab led to a general improvement in patient prognosis in several clinical trials [193]. For example, OC patients were shown to display a significantly improved progression-free survival upon bevacizumab administration [217]. Similarly, the combination of bevacizumab with a standard of care chemotherapeutic regimen led to improved progression-free survival both in NSCLC [218] and CRC [219,220]. Instead, discordant results were obtained with BC. Indeed, the addition of bevacizumab to neoadjuvant chemotherapy significantly improved the response of HER2-negative BC patients [221,222]. However, the addition of bevacizumab to paclitaxel did not prolong the overall survival of patients affected by metastatic BC [223].

### 6.2. Peripheral Nerves

**Figure 3:**
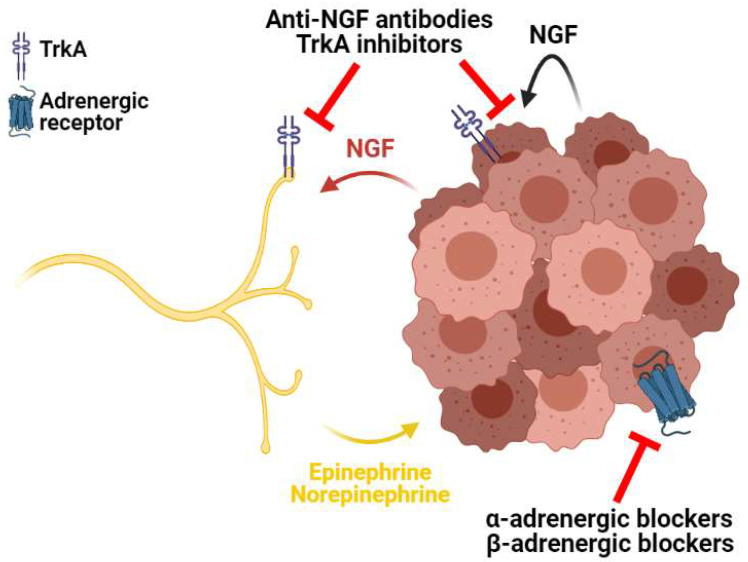
Therapeutic strategies aimed to hinder the peripheral nerve–tumor crosstalk. Anti-NGF antibodies and TrkA inhibitors impair both autocrine NGF signaling in the tumor and the NGF–TrkA interaction in peripheral nerves, thereby inhibiting axonogenesis. On the other hand, α- and β-adrenergic blockers counter both tumor-autonomous and nerve-mediated activation of adrenergic signaling, ultimately blocking cancer progression.

As we previously reported, axons are attracted within the TME by the local release of growth factors, including NGF [77]. Consistently, several studies showed that the inhibition of NGF has a direct effect on tumor growth, cancer progression, and the process of metastasis in different types of tumors [224] (Figure 3). Furthermore, NGF can exert an autocrine effect on tumor cells: indeed, NGF acts via the receptor Tyrosine kinase A (TrkA), and the binding activates several signaling cascades, including the PI3K-Akt and MAPK pathways, ultimately promoting cancer cell survival and proliferation [225]. On top of that, NGF and TrkA were shown to be overexpressed in BC [226], glioma [227], and other tumors [224] as compared to their normal counterparts. Therefore, TrkA represents a promising therapeutic target to counter tumor progression. Inhibition of NGF-mediated signaling using anti-NGF antibodies reduced BC growth in vivo and increased the numbers of cells that follow the apoptotic pathway [226]. In addition, a pioneer work on a panel of PC cell lines demonstrated that the TrkA competitive inhibitor CEP-751 induces tumor shrinkage in vivo without affecting normal prostate cells. Mechanistically, this effect is due to enhanced apoptosis in PC cells independently of their growth rate, differentiation, and metastatic potential [228]. Coherently, the use of CEP-751 on medulloblastoma and neuroblastoma significantly reduced tumor growth in vivo through the upregulation of the apoptotic pathway [229]. Similar results were obtained in PDAC, where TrkA chemical inhibitors impaired in vitro and in vivo growth via the inhibition of the MAPK pathway [230]. Furthermore, the neutralization of NGF with antibodies was reported to reduce BC angiogenesis in vivo via the inhibition of NGF-mediated VEGF release [231].

In addition, two independent works using in vitro co-cultures of PDAC cells and dorsal root ganglion neurons revealed that NGF knockdown abolishes both axon sprouting and tumor cell migration towards neurons [232,233], suggesting a fundamental role for the NGF–TrkA axis both in the axonogenesis process and in the metastatic progression of PDAC via PNI [233]. Concordantly, a recent study on GC demonstrated that chemical blockade of the NGF–TrkA axis via TrkA inhibitors leads to decreased axonogenesis in vivo, which, in turn, results in markedly reduced tumor growth [234]. Ultimately, an in vivo study on sarcoma bone metastases showed that the neutralization of NGF with anti-NGF antibodies significantly blocked the sprouting of sympathetic and sensory nerves in the bones, thereby inhibiting the development of cancer-related pain [235]. Interestingly, a clinical trial on patients with metastatic bone cancer revealed that the addition of the anti-NGF antibody tanezumab to a regimen of daily opioid treatment reduces chronic pain in patients with high pain and/or taking low doses of opioids [236].

Other studies tackled the neuronal signaling to the tumor to counter therapy resistance and tumor progression [237]. Pioneer works on cold stress—mimicked by reducing the mouse housing temperature—showed that the exposure to cold temperature raises the circulating levels of norepinephrine to activate adaptive thermogenesis [238,239]. In this respect, independent works on PDAC and CRC demonstrated that cold stress decreases the sensitivity to both radiotherapy [240] and chemotherapy [241] in vivo. In particular, the adrenergic signaling driven by cold stress significantly impaired the radiotherapy efficacy in CRC by both inducing resistance to radiation-mediated cell killing and by suppressing the antitumor immune response [240]. Furthermore, cold stress-induced β-adrenergic signaling decreases therapeutic sensitivity in vitro by increasing the levels of antiapoptotic proteins in PDAC. Coherently, in vivo administration of β-adrenergic blocker propranolol abrogates the resistance to Apo2L/TRAIL chemotherapy in PDAC [241]. In line with this, independent works on PDAC [242], NSCLC [243], HNSCC [244], BC [245], and neuroblastoma [246] revealed that in vitro administration of β-adrenergic blockers significantly sensitizes cancer cells to chemotherapy. Notably, the same effect was scored in GC [247], HNSCC [244], and NSCLC [243] cells exposed to radiotherapy in vitro. Interestingly, the antitumoral synergistic effect of β-adrenergic blockers with both chemotherapy and radiotherapy relied on the stimulation of the apoptotic pathway in cancer cells. Indeed, upon β-adrenergic blocker administration, cancer cells were shown to upregulate Bax and effector caspases and to expose phosphatidylserine in the outer leaflet of the plasma membrane [242,243,244,245,246]. Notably, PDAC patients in an adjuvant chemotherapy regimen displayed increased overall survival upon nonselective β-adrenergic blocker treatment as compared to the nonusers [59]. NSCLC patients were shown to display a better prognosis in terms of overall survival, distant metastasis-free survival, and progression-free survival when chemotherapy was combined with β-adrenergic blocker administration [243]. Besides eliciting chemo- and radiosensitivity, propranolol displays an antitumor effect per se through the induction of the apoptotic pathway in HCC [248] and multiple myeloma [249] in vitro. In line with these results, a recent clinical trial on hemangioma demonstrated that propranolol effectively fosters tumor regression upon six months of oral administration [250]. Similar results were obtained in BC [251], OC [252], and melanoma [253], where the β-adrenergic blocker treatment led to better patient prognosis. However, clinical studies on HNSCC [254], PC [255], and other cancer types [256] revealed that the administration of β-adrenergic blockers may exert a detrimental effect on patient prognosis, thus suggesting that the antitumoral effect of β-adrenergic blockers is largely dependent on the tumor type.

Similar to β-adrenergic receptors, α-adrenergic receptors can be bound by norepinephrine and epinephrine and represent intriguing targets for cancer therapy [242]. Indeed, α-adrenergic receptor antagonists doxazosin and terazosin were shown to inhibit both PC [257], OC [258], and BC [259] in vitro and in vivo growth via the activation of apoptosis. Concordantly, a recent in vitro study on a panel of cell lines belonging to PDAC, NSCLC, and GBM revealed that doxazosin administration fosters autophagy upregulation. Autophagy, in turn, mediates cell death and sensitizes cancer cells to the osimertinib chemotherapeutic agent [260]. Interestingly, a recent clinical trial on benign prostatic hyperplasia revealed that the administration of doxazosin significantly improves the clinical progression as compared to placebo [261], thus suggesting doxazosin as an exploitable therapy in PC [262]. However, the investigation of the doxazosin mechanism in vitro showed that the antitumor effects of this drug do not depend uniquely on α-adrenergic receptor inhibition [257,259]. Therefore, further studies are needed to clearly determine the role of alpha-adrenergic signaling in tumor progression and therapy.

## 7. Concluding Remarks

The interactions between tumoral cells and the surrounding TME are fundamental for cancer cells to progress throughout the various phases of tumor development. In this scenario, blood vessels and peripheral nerves gained increasing importance over time, as they are implicated in both cancer initiation and progression. In this review, we summarized the main characteristics of the tumor vasculature and innervation, including the plethora of signals that recruit them within the TME. Cancer cells attract blood vessels within the tumor mass to gain access to nutrient and oxygen supplies. On the other hand, the sprouting of nerve fibers in the TME significantly improves the fitness of cancer cells as neurotransmitters (most of all, epinephrine and norepinephrine) foster local tumor progression. In parallel, peripheral nerves and blood vessels were proven to have a role in metastasis spreading, as they provide both the molecular signals to ignite and the structural scaffold to allow metastasization. In this regard, increased MVD and PNI were clinically associated with poor patient prognosis. Therefore, the development of therapeutic strategies aimed to block the interaction between cancer cells and the tumor vasculature and innervation is becoming of paramount importance to improve patient survival. Several clinical trials demonstrated the importance of antiangiogenic drugs in reducing both local and distant tumor growth. Normalization of the tumor vasculature via antiangiogenic drugs (namely, TKIs and anti-VEGF antibodies) was shown to both sensitize tumoral masses to chemo- and radiotherapy and severely dampen both local and distant tumor progression. Further, both in vitro and pre-clinical in vivo studies revealed the prominent role of anti-NGF therapies in promoting tumor regression and PNI inhibition, thereby suggesting that NGF may become a relevant target for future anticancer therapies. Ultimately, inhibitors of α- and β-adrenergic receptors displayed significant antitumor activity in clinical trials. These results further corroborate the importance of adrenergic signaling in cancer progression and advocate α- and β-blockers as exploitable candidates in cancer therapy.
